# The impact of a sepsis performance improvement program in the emergency department: a before–after intervention study

**DOI:** 10.1007/s15010-022-01957-x

**Published:** 2022-11-17

**Authors:** Michiel Schinkel, Frits Holleman, Richarda Vleghels, Kayla Brugman, Milan L. Ridderikhof, Mahi Dzelili, Prabath W. B. Nanayakkara, W. Joost Wiersinga

**Affiliations:** 1 Center for Experimental and Molecular Medicine (CEMM), Amsterdam UMC Location University of Amsterdam, Meibergdreef 9, 1105 AZ Amsterdam, The Netherlands; 2 Department of Internal Medicine, Amsterdam UMC Location Vrije Universiteit Amsterdam, De Boelelaan 1117, Amsterdam, The Netherlands; 3 Amsterdam Institute for Infection and Immunity (AII), Amsterdam, The Netherlands; 4 Department of Internal Medicine, Amsterdam UMC University of Amsterdam, Meibergdreef 9, Amsterdam, The Netherlands; 5 Emergency Department, Amsterdam UMC University of Amsterdam, Meibergdreef 9, Amsterdam, The Netherlands; 6 Emergency Department, Amsterdam UMC Location Vrije Universiteit Amsterdam, De Boelelaan 1117, Amsterdam, The Netherlands; 7 Amsterdam Public Health, Amsterdam, The Netherlands

**Keywords:** Sepsis, Surviving sepsis campaign, Sepsis performance improvement program, Sepsis team

## Abstract

**Purpose:**

The latest Surviving Sepsis Campaign guidelines advocate that all hospitals use sepsis performance improvement programs. However, there is a limited evidence about how to structure such programs and what their potential impact is on sepsis management and outcomes in the emergency department (ED). In this study, we evaluated the implementation of a sepsis performance improvement program in the ED including a dedicated sepsis response team and analyzed the management and outcomes of sepsis patients before and after.

**Methods:**

We conducted a before–after interventional study in the ED of the Amsterdam University Medical Centers, the Netherlands. The sepsis performance improvement program included regular educational meetings, daily audits and weekly feedback, a screening tool, and a dedicated multidisciplinary sepsis response team. We studied all adult patients who presented to the ED with a suspected infection and a Modified Early Warning Score (MEWS) ≥ 3 during their stay. In the postintervention phase, these patients were seen by the sepsis team. Process-related and patient-related outcomes were measured between November 2019 and February 2020 (preintervention) and December 2021–May 2022 (postintervention).

**Results:**

A total of 265 patients were included in the primary study, 132 patients preintervention and 133 patients postintervention. The postintervention phase was associated with improvements in nearly all process-related outcomes, such as a shorter time to antibiotics (66 vs. 143 min; *p* < 0.001), increased number of lactate measurements (72.9 vs. 46.2%; *p* < 0.001), and improved completeness of documented MEWS scores (85.0 vs. 62.9%; *p* < 0.001). Except for an improvement in the number of immediate versus delayed ICU admissions (100% immediate vs. 64.3% immediate; *p* = 0.012), there was no improvement in the other patient-related outcomes such as 28 days mortality (14.3 vs. 9.1%; *p* = 0.261), during the postintervention phase.

**Conclusion:**

Our program stimulated physicians to make timely decisions regarding diagnostics and treatment of sepsis in the ED. Implementing the sepsis performance improvement program was associated with significant improvements in most process-related outcomes but with minimal improvements in patient-related outcomes in our cohort.

**Supplementary Information:**

The online version contains supplementary material available at 10.1007/s15010-022-01957-x.

## Introduction

Sepsis is a major global health problem defined as a life-threatening organ dysfunction caused by a dysregulated host response to infection [1, 2]. In 2017, the World Health Assembly of the World Health Organization declared sepsis a global priority and adopted a resolution to improve its prevention, diagnosis, and management [3]. With a recent estimate of 49 million sepsis cases each year, the global burden of sepsis may be more significant than previously anticipated [4, 5].

In the early 2000s, the Surviving Sepsis Campaign (SSC) was established to provide evidence-based guidelines for managing sepsis and septic shock [6]. The SSCs goal is to reduce sepsis morbidity and mortality worldwide, and its guidelines were most recently updated in 2021 [7, 8]. By bundling the guideline recommendations into core groups of clinical actions that should be performed within a specific timeframe, the SSC aims to facilitate implementation [8]. Several observational studies have shown that compliance with sepsis care bundles is associated with reduced mortality rates [9–13]. However, bundle adherence still remains a significant challenge, and non-compliance is especially prominent in the microbiological workup and timely administration of antibiotics in emergency departments (EDs) [14–16]. As the ED often represents a sepsis patient’s first interaction with the healthcare system, it is crucial to promptly initiate the appropriate care processes in this setting [16].

In response to the suboptimal compliance with sepsis guidelines, hospitals and healthcare organizations have initiated sepsis performance improvement programs. These initiatives often include interventions such as educational programs, screening tools, or changes in sepsis care pathways (e.g., activating dedicated sepsis response teams) [17]. Performance improvement programs have been associated with better adherence to SSC or local sepsis guidelines and decreased mortality rates [18]. The latest SSC guideline thus advocates that all hospitals and health systems implement sepsis performance improvement programs [8].

Although the use of sepsis performance improvement programs is now recommended, there is limited evidence on how these programs should optimally be structured [17, 18]. Furthermore, their potential impact on the ED population is relatively unknown, as most studies target the intensive care unit (ICU) population [17, 18]. In this study, we prospectively evaluate the implementation of a multidisciplinary sepsis response team and performance improvement program in our ED and analyze the management and outcomes of sepsis patients before and after.

## Methods

We conducted a before–after intervention study in the ED of the Amsterdam University Medical Centers—location VUmc, in the Netherlands. The Medical Ethics Review Committee waived the review of this study as it was a quality improvement project within regular care (IRB number: IRB00002991; case:19.449). Study outcomes were measured between November 2019 and February 2020 (preintervention phase) and December 2021–May 2022 (postintervention phase), while the period in between March 2020 and November 2021 was used to implement all aspects of the sepsis performance improvement program appropriately (implementation phase). Patients were sent a letter to opt out of the use of their data for this project. We adhere to “The Strengthening the Reporting of Observational Studies in Epidemiology (STROBE) Statement: guidelines for reporting observational studies” [19].

### Study population

We studied all adult patients (18 years and older) who presented to the ED with a suspected infection and a Modified Early Warning Score (MEWS) ≥ 3 during their stay. We used the MEWS with a cut-off at three to screen for sepsis, following the guidelines from the Dutch Federation of Medical Specialists (FMS), National Patient Safety Programme (VMS), and the SSC guideline [8, 20, 21]. Patients were excluded from the study if they were pregnant, SARS-CoV-2 positive before arriving at the ED, or when they opted out of participating.

### Intervention

**Fig. 1:**
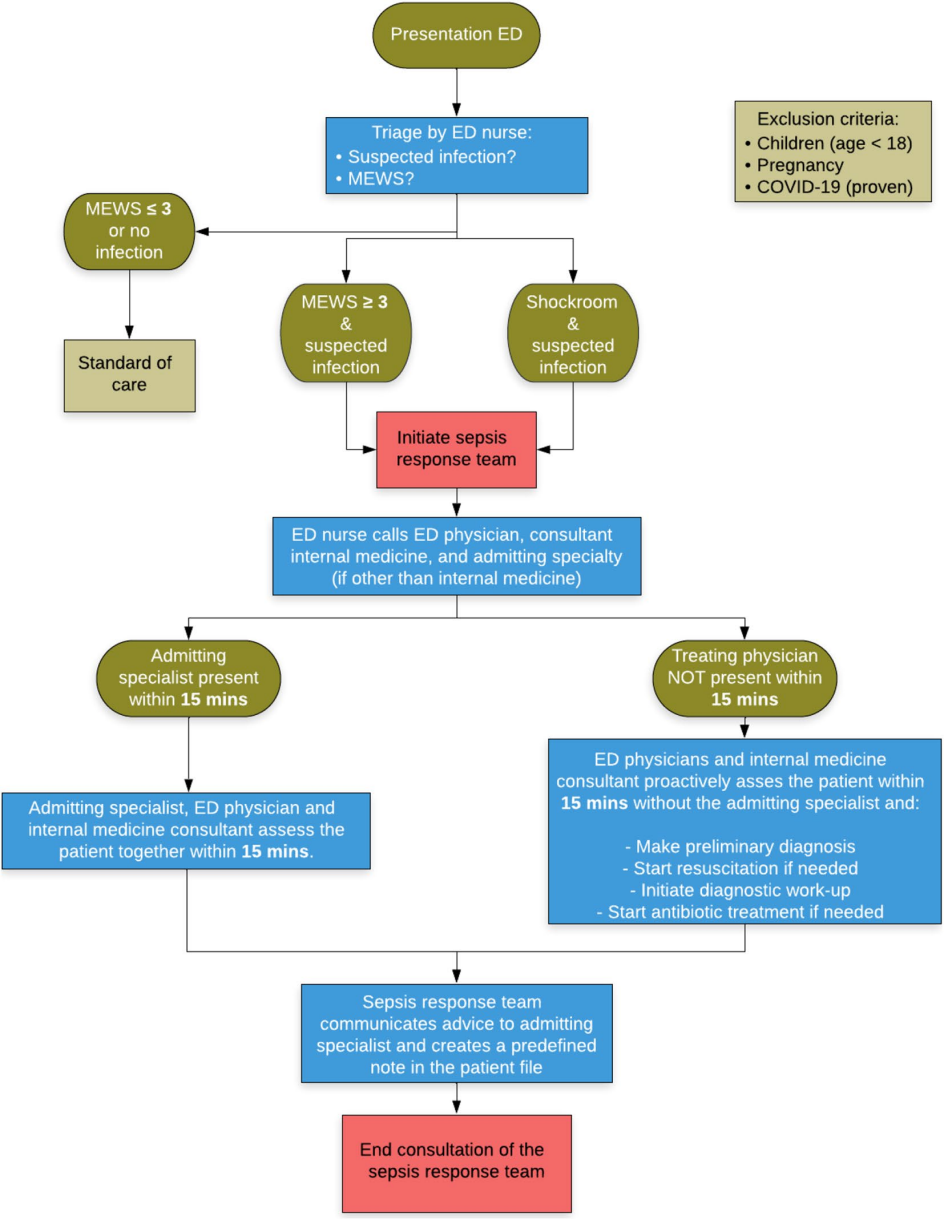
The flowchart for the activation of the sepsis response team as implemented in the ED of the Amsterdam UMC. *ED* Emergency Department; *MEWS* Modified Early Warning Score

The preintervention measurements were performed before our sepsis performance improvement initiative was started, and the hospital’s standard care was provided to all patients with suspected infections. The MEWS was already part of the hospital’s standard screening procedures during this period, although compliance with these procedures was variable. Afterwards, we introduced several interventions: regular educational meetings at morning handovers; standardized sepsis team notes and order sets in the Electronic Health Record (EHR) system; daily audits and weekly feedback; the systematic use of a screening tool (MEWS); and most importantly, the introduction of a multidisciplinary sepsis response team. During the postintervention phase, the sepsis team was active in the ED 24 h a day, 7 days a week. An ED nurse on duty alerted the sepsis team when a patient was identified as having a suspected infection and had a MEWS ≥ 3 during the ED stay. The multidisciplinary sepsis response team consisted of the on-call physician from the following departments: emergency medicine, internal medicine, and the admitting specialty (e.g., surgery, urology, neurology, etc.). The team aimed to assess all patients within 15 min after a MEWS ≥ 3 was recorded in the ED. Following the assessment of the patient, the team advised the on-call physician of the admitting specialty regarding the diagnostic workup and treatment based on the local protocol, which was adapted according to the SSC guidelines. The Amsterdam UMC follows the national antibiotic sepsis guidelines of the Dutch Working Party on Antibiotic Policy (SWAB; https://swab.nl/en/swab-guidelines), which did not change during the study period. The workflow, which focused on collaboration and shared responsibility across specialties, was created with input from emergency and intensive care physicians, internal medicine specialists, radiologists, and patient representatives. The complete sepsis team workflow is visually presented in Fig. 1.

### Data and outcomes

To study the impact of the implementation of our sepsis team, we looked at two distinct data categories: process-related and patient-related outcomes. All study data were collected from the EHR. The base dataset included patient characteristics such as age, sex, and comorbidities. For process-related outcomes, we collected data on aspects of the diagnostic workup (e.g., blood cultures taken, lactate measurements) and the treatment strategy (e.g., administration of antibiotics and fluids). For patient-related outcomes, we extracted data such as ED length of stay, admission rates, and mortality rates. Furthermore, we studied the number of patients directly admitted to the ICU from the ED (immediate ICU admission) compared with those who were first admitted to regular wards and further transferred to the ICU within the first 48 h (delayed ICU admission).

Besides the outcome measures, we also investigated the specific infections and diagnoses in the study cohort. The type of infection and final diagnosis were assessed by a clinical adjudication committee consisting of an experienced ED nurse (RV), a last-year medical student (KB), and a medical doctor (MS), and based on all microbiology results (including all culture results, polymerase chain reaction testing, etc.) and the medical notes in the EHR. Furthermore, sepsis or the progression to sepsis in the first 72 h was assessed based on the Sepsis-3 definition, using the Sequential Organ Failure Assessment (SOFA) score [1]. The SOFA score uses PaO2/FiO2 ratios to determine respiratory dysfunction. These ratios require arterial blood gas measurements, which are infrequently performed in EDs. Therefore, we used the SpO2/FiO2 ratio and corresponding cut-offs as a proxy for the PaO2/FiO2 ratio, as previously described [22, 23]. For all SOFA items, the worst value of the day was used. When an item was not measured on a given day, the score for that part of the SOFA was zero.

### Statistical analysis

We hypothesized that the ED length of stay would be the most likely patient-related outcome that could be impacted by introducing a sepsis team. To detect a statistically significant change in the ED length of stay of at least 30 min, from a retrospective baseline of approximately 228 min and a standard deviation of 87 min, we needed to include a total of 266 patients (133 patients per phase) to find a difference with a power of 80%.

Descriptive statistics were used to assess all variables. Continuous variables were described by their median and interquartile range (IQR) and compared between the preintervention and postintervention phase using a *t* test or Wilcoxon–Mann–Whitney test when appropriate considering the distribution. Categorical variables were described using frequencies and percentages, and differences were calculated using the Chi-square test.

During the postintervention phase, the sepsis team did not cover all eligible patients suspected of infection with an elevated MEWS score. Therefore, a comparison between the postintervention groups “sepsis team activated” and “sepsis team not activated” was conducted to better evaluate the sepsis team’s effect. Group differences were further examined with a multivariable linear or logistic regression to establish whether these differences could be explained by differences in baseline characteristics between the groups. The outcomes were adjusted for age, comorbidity index, MEWS in the ED, and “do not resuscitate” policies.

Statistical significance was defined as a two-tailed *p* value < 0.05. The analyses were performed using R (version 4.2.1) and the R packages: “tidyverse”, “ggplot2”, “ggpubr”, “naniar”, and “tableone” [24].

## Results

A total of 265 patients were included in the primary study, 132 patients preintervention and 133 postintervention. The median age was 68 years (interquartile range [IQR]: 56–77), and 61.5% of the patients were male. The median MEWS score in the ED was 4 (IQR: 4–6), and the Charlson Comorbidity Index was 5 (IQR: 2–7). Baseline characteristics are provided in Table 1. The most common site of the suspected Infection in the ED was the respiratory tract in both the pre- and post-intervention phases. However, respiratory tract infections were relatively more common in the preintervention phase. e-Fig. 1 of the supplementary appendix shows the distribution of the suspected infection sites and the most likely infection type at discharge in the different phases.

**Table 1:** Characteristics of patients presenting to the emergency department with a suspected infection and modified early warning score ≥ 3

Characteristics	Totals (*n* = 265)	Preintervention (*n* = 132)	Postintervention (*n* = 133)	*p* value
Age, year	68 (56–77)	69 (57.5–77)	67 (56–76)	0.574
Sex, male	163 (61.5%)	80 (60.6%)	83 (62.4%)	0.861
Charlson comorbidity Index	5 (2–7)	5 (2–7)	5 (3–7)	0.367
MEWS in ED	4 (3–6)	4 (3–5)	5 (4–6)	< 0.001
Do not resuscitate orders	89 (33.7%)	38 (29.0%)	51 (38.3%)	0.275
Diagnostic workup and treatment				
Complete MEWS recorded	196 (74.0%)	83 (62.9%)	113 (85.0%)	< 0.001
Time to MEWS ≥ 3, minutes	17 (9–30)	19 (10–28.75)	16 (8–30)	0.315
Blood culture taken in the ED	209 (78.9%)	93 (70.5%)	116 (87.2%)	< 0.001
Time to blood culture, minutes	28 (14–65)	42 (20–126)	24 (9.75–49.75)	< 0.001
Antibiotics administered in the ED	179 (67.5%)	73 (55.3%)	106 (79.7%)	< 0.001
Time to first antibiotics, minutes	95 (43–181)	143 (91–250)	66 (40–123.75)	< 0.001
Antibiotics administered and blood culture taken in the ED	159 (60.0%)	62 (47.0%)	97 (72.9%)	< 0.001
Blood cultures before antibiotics	138 (86.8%)	52 (83.9%)	86 (88.7%)	< 0.001
Lactate measurement	158 (59.6%)	61 (46.2%)	97 (72.9%)	< 0.001
Repeat measurement of lactate	52 (32.9%)	10 (16.4%)	42 (43.3%)	< 0.001

A comparison is made between the preintervention and postintervention phases of the implementation of the sepsis improvement program in the ED

*ED* Emergency Department, *MEWS* Modified Early Warning Score. Data are presented with no. (%) or median (interquartile range). The results of the indented characteristics are calculated based on the number of patients in the main group of that characteristic

### Before–after comparison

#### Process-related

First, we studied the effect of our sepsis performance improvement program on process-related outcomes. During the postintervention phase, the complete MEWS assessment (all items recorded) was performed more frequently (85.0 vs. 62.9%; *p* < 0.001), but the time to the first recorded MEWS (≥ 3) was similar (16 vs. 19 min; *p* = 0.315). Blood cultures were drawn significantly more often during the postintervention phase (87.2 vs. 70.5%; *p* < 0.001), and the time until blood cultures were drawn was lower (24 vs. 42 min; *p* < 0.001). Antibiotics were administrated in the ED in more cases during the postintervention phase (79.7 vs. 55.3%; *p* < 0.001), and the time to antibiotics was significantly lower (66 vs. 143 min; *p* < 0.001). Lactate measurements were performed more often (72.9 vs. 46.2%; *p* < 0.001), and repeat measurements were also performed more frequently (43.3 vs. 16.4%; *p* < 0.001). Taken together, these results show that the implementation of our sepsis performance improvement program was associated with improvements in most process indicators.

#### Patient-related outcomes

Next, we examined whether the process-related improvements translated into improved patient-related outcomes. Our main outcome parameter was the length of stay in the ED, which was similar in the postintervention phase compared to the preintervention phase (283 vs. 287 min; *p* = 0.983). Hospital admission rates were also similar in both phases (88.0 vs. 83.3%; *p* = 0.367). There were no significant differences in the number of ICU admissions (16.5 vs. 10.6%; *p* = 0.353) or the length of stay in the ICU (2 vs. 2.5 days; *p* = 0.830). However, the number of immediate ICU admissions from the ED was significantly higher in the postintervention group compared to the preintervention group (100% immediate vs. 64.3% immediate; *p* = 0.012). However, the hospital length of stay was significantly longer in the postintervention phase (5 days vs. 4 days; *p* = 0.033). There were no significant differences regarding 28 days mortality (14.3 vs. 9.1%; *p* = 0.261) or 28 days hospital readmissions (10.5 vs. 17.4%; *p* = 0.096) between the groups. An overview of the patient-related outcomes is provided in Table 2.

**Table 2:** Outcomes of patients presenting to the emergency department with a suspected infection and modified early warning score ≥ 3

Outcome	Totals (*n* = 265)	Preintervention (*n* = 132)	Postintervention (*n* = 133)	*p* value
ED length of stay, minutes	286 (221–407)	287 (224–407)	283 (221–409)	0.983
Hospital admission	227 (85.7%)	110 (83.3%)	117 (88.0%)	0.367
Hospital length of stay, days	5 (3–9)	4 (2–9)	5 (3–10)	0.033
ICU admission	36 (13.6%)	14 (10.6%)	22 (16.5%)	0.456
Immediate ICU admission from ED	31 (86.1%)	9 (64.3%)	22 (100%)	0.012
ICU length of stay, days	2 (1.75–7.75)	2.5 (2–4)	2 (1.25–11.50)	0.704
28 days mortality	31 (11.7%)	12 (9.1%)	19 (14.3%)	0.261
28 days readmission	37 (14.0%)	23 (17.4%)	14 (10.5%)	0.096
SOFA score ≥ 2 within 72 h	193 (72.8%)	95 (72.0%)	98 (73.7%)	0.861

A comparison is made between the preintervention and postintervention phases of the implementation of the sepsis improvement program in the ED

*ED* Emergency Department, *ICU* Intensive Care Unit, *SOFA* Sequential Organ Failure Assessment. Data are presented with no. (%) or median (interquartile range). The results of the indented characteristics are calculated based on the number of patients in the main group of that characteristic

To further investigate whether the implementation of our sepsis team may have impacted mortality, we created a logistic regression model to explain 28 days mortality by the sepsis team implementation, adjusted for age, MEWS, comorbidity index, and “do not resuscitate” (DNR) policy. The odds ratio (OR) for 28 days mortality in the postintervention phase was 1.24 (95% confidence interval (CI): 0.54–2.92; *p* = 0.611). In this model, only the DNR policy was significantly associated with 28 days mortality with an OR of 6.70 (95% CI 2.53–20.12; *p* < 0.001). We also created a linear regression model to examine whether the significantly longer length of stay in the hospital in patients seen by the sepsis team could be explained by differences in the baseline characteristics. After adjustment for age, MEWS, comorbidity index, and DNR policy, the use of the sepsis team was no longer associated with a prolonged hospital stay (*p* = 0.171). In this model, only the MEWS in the ED was significantly associated with the hospital length of stay (*p* < 0.001). Overall, these results show that, except for an increase in the percentage of direct ICU admissions from the ED compared to delayed ICU admissions, there were no meaningful differences in patient-related outcomes before and after the implementation of our sepsis performance improvement program.

#### Infection and sepsis

We also investigated the type of patients identified through our intervention program. The number of patients who fulfilled the sepsis criteria during the first 72 h of admission was calculated using the SOFA score. We found no differences in the number of patients fulfilling the Sepsis-3 criteria (73.7 vs. 72.0%; *p* = 0.395) in the preintervention and postintervention phases. When we looked at the most likely etiology of the infections at discharge (based on all microbiology results and medical notes), we observed different distributions of causative agents before and after the implementation of the sepsis team. As shown in e-Fig. 1, the most common preimplementation infection type was viral (non-COVID-19; predominantly influenza). After the implementation, the majority of infections were bacterial.

### Comparison between the postintervention groups (post–post)

Since there was no complete compliance with the sepsis team activation, we could study an additional cohort of control patients in the postintervention phase for whom the sepsis team was not activated. During the postintervention phase, the sepsis team was activated for 133/207 (64%) of all eligible patients. A comparison of the baseline characteristics of postintervention groups in which the sepsis team was, or was not activated, is shown in e-Table 1 of the supplementary appendix.

The patients for whom the sepsis team was activated had similar Charlson Comorbidity Index scores (5 vs. 4; *p* = 0.078) but a higher MEWS score on presentation (5 vs. 4; *p* = 0.007). When the sepsis team was activated, MEWS scores were recorded completely in more cases (85.0 vs. 66.2%; *p* = 0.003). A similar number of blood cultures was performed (87.2 vs. 86.5%; *p* = 1.000), but they were performed faster when the sepsis team was activated (24 vs. 43.5 min; *p* = 0.009). Antibiotics were administered more frequently (79.7 vs. 63.5%; *p* = 0.017), and the time to antibiotic treatment was lower (66 vs. 126 min; *p* = 0.001) in those patients for which the sepsis team was activated compared to those patients for which the sepsis team was not activated. Activation of the sepsis team resulted in a higher number of lactate measurements (72.9 vs. 50.0%; *p* = 0.002), while the rates of repeat measurement lactate levels were statistically comparable (43.3 vs. 24.3%; *p* = 0.085).

Except for an increased number of ICU admissions directly from the ED (*p* = 0.033), we observed no significant differences in patient-related outcomes such as ED length of stay, admission rates, or mortality rates, as further highlighted in e-Table 2 of the supplementary appendix. However, the postintervention group in which the sepsis team was activated had significantly more cases that fulfilled the sepsis criteria within the first three days of admission (73.7 vs. 51.4%; *p* = 0.002). Taken together, the post–post comparison reinforces that the sepsis improvement program is associated with improved process-related outcomes, including a 50% lower time to antibiotics, though there were few observable patient benefits.

## Discussion

In this study, we found that the implementation of a sepsis performance improvement program in the ED including the use of a specialized multidisciplinary sepsis response team resulted in better identification of sepsis, an improved diagnostic process, and a > 50% reduction in time to antibiotic treatment in suspected sepsis patients. However, these improved process-related outcomes did not translate into improvements in length of stay, admission rates, or mortality rates. The only patient-related outcome which improved was the number of immediate versus delayed ICU admissions.

Implementing our sepsis performance improvement program was associated with various improved process-related outcomes. The MEWS score, which already was the preferred screening tool according to hospital policy in the preimplementation phase, was recorded completely in significantly more cases. Interestingly, the MEWS recordings were also more complete when comparing postimplementation patients for whom the sepsis team was or was not activated (post–post comparison). This indicates that the triage nurses indeed linked the use of the MEWS as a screening tool to the sepsis performance improvement program but did not use it when they did not consider the patient as a potential sepsis case. In addition, the number of lactate measurements also significantly increased after implementation and remained significant in the post–post comparison. The SSC guideline recommends using both sepsis screening tools (including MEWS) and lactate measurements, and we thus show increased SSC guideline adherence in these instances [8]. Regarding the workup with blood cultures, we found mixed results. Though there seems to be an increase in the number of blood cultures performed when comparing preimplementation and postimplementation patients, the post–post comparison does not reinforce this effect. ED nurses seem to have been more inclined to draw blood cultures postintervention, irrespective of whether or not the patient was seen by the sepsis team. This could be due to the attention given to blood cultures in the educational meetings as part of the sepsis improvement program, but it could also be a reflection of the higher rate of bacterial infections in the post-phase, as seen in Fig. 1B of the supplementary appendix. In line, sepsis team utilization was associated with a decrease in time to blood culture draws when compared to both control groups.

The implementation of the sepsis team was associated with a considerable reduction in the time to the first administration of antibiotics. Although the benefits of early antibiotics for all sepsis patients remain debatable, there are specific subgroups of patients who may experience benefits [25–29]. The sepsis team also started antibiotic treatment in the ED in significantly more cases than in the preimplementation phase or in the postimplementation patients when no sepsis team was involved. This indicates that the improved recognition of sepsis may have led to an increased use of antibiotics. Notably, not all patients seen by the sepsis team were treated with antibiotics in the ED. In cases with a relatively low probability of sepsis or shock, the latest SSC guidelines suggest conducting a time-limited investigation first and only initiating antimicrobial therapy when the concern for infection persists [8].

Despite many improved process-related outcomes, we found only a single patient-related outcome that improved after implementing the sepsis team in the ED, which contrasts with previous literature [16, 30, 31]. Viale et al*.* found that their infectious diseases team improved SSC guideline adherence in a general ED in Italy [16]. Their pre–post comparison including 382 (195 vs. 187) severe sepsis and septic shock patients with a high median age of 82 years (IQR 70–88) showed that the infectious diseases team implementation was associated with higher rates of lactate measurements (90 vs. 76%; *p* < 0.001) and blood cultures before antibiotics (58 vs. 42%; *p* < 0.001). The time to first antibiotic treatment did not significantly decrease (154 vs. 169 min; *p* = 0.42). Interestingly, the all-cause 14 days mortality was significantly lower in univariate and multivariate analyses (29 vs 39%; *p* = 0.02), but the 30 days all-cause mortality was not (37 vs. 45%; *p* = 0.102). Arabi et al*.* implemented a multifaceted intervention similar to ours, including a sepsis response team, in their ED in Saudi Arabia [31]. In that postintervention cohort of 699 patients, most process-related and patient-related outcomes improved significantly. For example, the percentage of patients receiving antibiotics within 3 h improved (89.4 vs. 67.7%; *p* < 0.001), and the hospital mortality rate was lower (16.9% vs. 47.7; *p* = 0.003). A recent publication by Simon et al*.* also shows improvements associated with a sepsis team implementation in the ED of a tertiary hospital in the United States. The pre–post analysis among 863 patients (393 vs. 470) showed that the time to antibiotics was reduced (81 vs. 107 min; *p* < 0.001), just as the in-hospital mortality (15.1 vs. 28.2%; *p* < 0.001). A notable difference with all of these cohorts is that their preintervention mortality rates of 45%, 48%, and 28% were much higher than in our cohort (9.1%). This finding is not completely unexpected since other Dutch studies and various international sepsis studies in the ED setting have also reported relatively low sepsis mortality rates [26, 27]. Furthermore, the aim of this sepsis performance improvement program was to screen for sepsis and detect and treat it early. Mortality rates in such a screening cohort will be lower than in cohorts looking only at definite and severe sepsis cases. We may argue that sepsis performance improvement programs are more likely to improve mortality at those higher mortality rates or that establishing a significant mortality benefit is at least easier in such a population. Still, we would have expected to find other improvements in patient-related outcomes through our intervention, especially a shorter length of ED stay. Unfortunately, overcrowding of the ED and exit blocks toward the hospital wards due to staff shortages in our postintervention phase made it challenging to transfer patients to the wards [32, 33]. During the extraction of data from the EHR system, the study team had the impression that patients were ready for hospital admission earlier when they were seen by the sepsis team, but this could not be reflected in shorter ED stays due to the logistical constraints. This hypothesis is further supported by the fact that we were able to show a significant improvement in the number of direct versus delayed ICU admissions. Delayed ICU admission (patients who will eventually need an ICU admission but are first admitted to the regular ward) is an independent risk factor for sepsis mortality, but none of the patients seen by the sepsis team were being admitted to the ICU with a delay [34, 35]. This suggests that our intervention helped bring together the experts needed to make the most appropriate and timely decision about where the patient needed to go next.

To fully understand the results of this study, it is essential to acknowledge the role of the COVID-19 pandemic. Shortly after our preimplementation measurements, the SARS-CoV-2 virus emerged [36]. The pandemic put unprecedented pressure on healthcare workers and hospitals, which caused a significant delay in the implementation of our sepsis team [37]. Consequently, a near 2 years interval was needed before the postimplementation measurements could be performed. Even then, the healthcare system, and certainly the ED, continued to operate under high pressure, which led to imperfect compliance rates with the sepsis team activation. In the meantime, the national report on infectious diseases in the Netherlands and several international publications showed that the distribution of infectious agents had changed, with, for example, a much lower prevalence of influenza [38–40]. Our study observed similar changes, where influenza was much less prevalent in the postimplementation phase, while bacterial infection rates were higher. Of note, proven COVID-19 cases at presentation were excluded. Although sepsis guidelines are created for a heterogeneous group of patients with all types of infections, the results of our before-after comparison may have been influenced by the COVID-19 pandemic-induced changes in the causative agents of sepsis in our population. Bacterial infections seemed to have been much more prevalent during the postintervention phase. Fortunately, the imperfect compliance rates with the sepsis team activation led us to have an additional cohort of patients from the postimplementation phase in whom no sepsis team was activated and who could serve as an unexpected but essential second control group.

Besides the potential confounding through COVID-19, several other limitations of this study must be addressed. First and foremost, this study was powered to detect a difference in the length of ED stay after implementing a sepsis team. In hindsight, this was an unattainable result due to the logistical constraints (e.g., exit blocks) discussed above. Second, the compliance rate with the sepsis team activation was only 64%. Consequently, selection bias may have been introduced since the ED nurses may have had an unconscious bias to activate the sepsis team only in more severe cases, in whom the diagnostic workup and start of treatment would already happen more timely. Fortunately, we could negate part of this confounding by comparing the pre–post results to the post-post comparison. Still, the fact that patients in the postintervention phase may have been more severely ill and less likely to survive compared with the preintervention phase should be considered when interpreting these results. Interestingly, it seems that the ED nurses could identify the patients with a higher likelihood of having sepsis, as the rate of progression to sepsis was significantly higher in postimplementation patients who were seen by the sepsis team. This finding supports our approach of implementing a sepsis response workflow based on the SSC recommendations but with relative flexibility to maneuver according to clinical judgment. Lastly, the before–after study design has its inherent limitations, such as time-related changes in populations and standards of care. A large (stepped wedge cluster) randomized trial is needed to fully understand the value of sepsis teams and sepsis performance improvement programs in general. Given the limited evidence for the benefits and the proper structure of a sepsis team, we did not have the support base to conduct such a trial. We hope our current work helps create the urgency for this type of study.

In conclusion, implementing our sepsis performance improvement program in the ED was associated with a number of improvements in process-related outcomes but minimal improvements in patient outcomes. The program stimulated physicians to make collaborative and timely decisions regarding diagnostics and treatment of sepsis. The workflow allowed them to incorporate their clinical judgment while still reinforcing the essential elements of sepsis care.

## Supplementary Information

Below is the link to the electronic supplementary material.

Supplementary file1 (DOCX 148 KB)

## Data Availability

The datasets used and/or analyzed during the current study are available from the corresponding author on reasonable request.
